# *KRAS* mutation analysis: a comparison between primary tumours and matched liver metastases in 305 colorectal cancer patients

**DOI:** 10.1038/bjc.2011.26

**Published:** 2011-03-01

**Authors:** N Knijn, L J M Mekenkamp, M Klomp, M E Vink-Börger, J Tol, S Teerenstra, J W R Meijer, M Tebar, S Riemersma, J H J M van Krieken, C J A Punt, I D Nagtegaal

**Affiliations:** 1 Department of Medical Oncology, Radboud University Nijmegen Medical Centre, PO Box 9101, Nijmegen, HB 6500, The Netherlands; 2 Department of Pathology, Radboud University Nijmegen Medical Centre, PO Box 9101, Nijmegen, HB 6500, The Netherlands; 3 Department of Epidemiology, Biostatistics and Health Technology Assessment, Radboud University Nijmegen Medical Centre, PO Box 9101, Nijmegen, HB 6500, The Netherlands; 4 Department of Pathology, Rijnstate hospital, Arnhem, The Netherlands; 5 Laboratory of Pathology East Netherlands, Enschede, The Netherlands

**Keywords:** *KRAS* mutation, colorectal cancer, liver metastases, concordance

## Abstract

**Background:**

*KRAS* mutation is a negative predictive factor for treatment with anti-epidermal growth factor receptor antibody in metastatic colorectal cancer (CRC). *KRAS* mutation analysis is usually performed on primary tumour tissue because metastatic tissue is often not available. However, controversial data are available on the concordance of test results between primary tumours and corresponding metastases. We assessed the concordance of *KRAS* mutation status in a study of 305 primary colorectal tumours and their corresponding liver metastases.

**Methods:**

Patients with histologically confirmed CRC who underwent surgical resection of the primary tumour and biopsy or surgical resection of the corresponding liver metastasis were included. *KRAS* mutation analysis was performed for codons 12 and 13.

**Results:**

*KRAS* mutation was detected in 108 out of 305 primary tumours (35.4%). In 11 cases (3.6%), we found a discordance between primary tumour and metastasis: 5 primary tumours had a *KRAS* mutation with a wild-type metastasis, 1 primary tumour was wild type with a *KRAS* mutation in the metastasis, and in 5 cases the primary tumour and the metastasis had a different *KRAS* mutation.

**Conclusion:**

We observed a high concordance of *KRAS* mutation status of 96.4% (95% CI 93.6–98.2%) between primary colorectal tumours and their corresponding liver metastases. In only six patients (2.0% 95% CI 0.7–4.2%), the discordance was clinically relevant. In this largest and most homogenous study to date, we conclude that both primary tumours and liver metastases can be used for *KRAS* mutation analysis.

Recent advances in specific signalling pathways of cancer cells have introduced targeted therapy into treatment regimes for patients with metastatic colorectal cancer (CRC) (Tol and Punt, 2010). Cetuximab and panitumumab are monoclonal antibodies that bind to the extracellular domain of the epidermal growth factor receptor (EGFR). They inhibit ligand-induced stimulation of several intracellular signalling pathways, such as RAS/RAF/MAPK and phosphoinositide-3 pathway, which results in decreased stimulation of cell cycle progression, proliferation, angiogenesis, and stimulation of apoptosis (Scaltriti and Baselga, 2006). The *KRAS* oncogene is currently the most relevant molecular biomarker that predicts the response to EGFR-targeted therapy in CRC. An oncogenic mutation in *KRAS* leads to constitutive activation of the RAS/RAF signalling pathway independent from EGFR activation by binding of the ligand (Benvenuti *et al*, 2007). *KRAS* mutations occur in approximately 38% of colorectal tumours and involve codon 12 and 13 in >96% of cases (Oliveira *et al*, 2004). Metastatic CRC patients with tumours harbouring a *KRAS* mutation are resistant to treatment with anti-EGFR antibodies, showing lower response rates, decreased progression-free survival, and overall survival compared with patients with *KRAS* wild-type tumours (Karapetis *et al*, 2008; Tol *et al*, 2009; Van Cutsem *et al*, 2009). Therefore, the European Medicines Agency and the Food and Drug Administration have restricted the use of anti-EGFR antibodies in metastatic CRC to patients with *KRAS* wild-type tumours.

Cetuximab and panitumumab have shown efficacy both as monotherapy (Amado *et al*, 2008; Karapetis *et al*, 2008) and in combination with chemotherapy (Tol *et al*, 2009; Van Cutsem *et al*, 2009) in patients with *KRAS* wild-type metastatic CRC. Nevertheless, even among patients with *KRAS* wild-type tumours, the majority of patients do not respond to anti-EGFR therapy. Efficacy of anti-EGFR therapy was suggested to be further restricted to patients with *BRAF* wild-type tumours (Di Nicolantonio *et al*, 2008). An additional explanation for the suboptimal response rates to anti-EGFR antibodies in patients with *KRAS* wild-type tumours is discordance of *KRAS* mutation status between primary colorectal tumours and corresponding metastases. In the early dissemination model, tumour cells depart the primary lesion before the acquisition of a fully malignant phenotype to undergo new mutations and metastatic growth at a distant site (Klein, 2009). According to this model, a discordance in mutation status between primary tumours and metastases may occur, and as a consequence the mutation status of the primary tumour might not be adequate to predict the response of metastases to anti-EGFR treatment.

Current data on the concordance in *KRAS* mutation status between primary colorectal tumours and metastases are conflicting. Five studies showed a 100% concordance of *KRAS* mutation status in primary CRC and corresponding metastases (Losi *et al*, 1992; Suchy *et al*, 1992; Zauber *et al*, 2003; Weber *et al*, 2007; Etienne-Grimaldi *et al*, 2008). In contrast to these data, others have reported a discordance of *KRAS* mutation status in primary tumours and metastatic sites, with an overall discordance observed in 4–32% of the patients (Oudejans *et al*, 1991; Al-Mulla *et al*, 1998; Albanese *et al*, 2004; Oliveira *et al*, 2007; Artale *et al*, 2008; Santini *et al*, 2008; Cejas *et al*, 2009; Garm Spindler *et al*, 2009; Loupakis *et al*, 2009; Molinari *et al*, 2009; Perrone *et al*, 2009; Baldus *et al*, 2010; Italiano *et al*, 2010). These controversial results are probably due to the fact that these studies were underpowered with a small number of patients, and included a wide variety of metastatic sites. Therefore, it is still uncertain whether the evaluation of *KRAS* mutation status in the most commonly available primary tumour correctly reflects the *KRAS* mutation status of corresponding metastasis. This is highly relevant given the large number of CRC patients as well as the potential toxicity and costs of anti-EGFR therapy.

We assessed the concordance in *KRAS* mutation status in primary tumours and their corresponding liver metastases in an adequately powered study of 305 CRC patients.

## Patients and methods

### Patient selection

Patients with histologically confirmed CRC who underwent surgical resection of the primary tumour and biopsy or surgical resection of the corresponding liver metastasis were included in this analysis. Results were obtained from archived material of three large pathology laboratories and from material collected from the CAIRO2 study, a large multicentre trial of the Dutch Colorectal Cancer Group (Tol *et al*, 2009).

In patients with a discordance of *KRAS* mutation status between the primary tumour and metastasis, additional blocks of the primary tumour were obtained to exclude heterogeneity within the tumour. Lymph node metastases present at the time of diagnosis were also acquired in these patients.

### Tumour DNA preparation

Formalin-fixed paraffin-embedded tissue blocks were cut at 4 *μ*m thickness and stained with haematoxylin and eosin (HE). The presence of tumour tissue was marked by a pathologist. Subsequently the blocks were cut at 20–40 *μ*m thickness and micro dissected for DNA extraction. Tumour tissue was dissolved in 200 *μ*l lysis buffer (QIAamp DNA Micro Kit, Qiagen, Venlo, The Netherlands) and incubated with proteinase K overnight at 56 °C for two nights. DNA was extracted according to the manufacturer's protocol (QIAamp DNA Micro Kit, Qiagen), and DNA concentration was determined at 260 nm using the Nanodrop 26 ND-1000 spectrophotometer (Nanodrop Technologies Inc., Wilmington, NC, USA).

### *KRAS* mutation analysis

For *KRAS* mutation analysis, exon 2 (codon 12 and 13) was amplified using a 50 *μ*l reaction mixture containing 0.2 *μ*m forward (5′-TGTAAAACGACGGCCAGTAGGCCTGCTGAAAATGACTG-3′) and reverse (5′-CAGGAAACAGCTATGACCTGGATCATATTCGTCCACAAAA-3′) primers (Invitrogen, Breda, The Netherlands); dATP, dCTP, dGTP and dTTP (GE Healthcare, Zeist, The Netherlands) at 0.2 mM each; 50 mM KCl; 10 mM Tris-HCl (pH 8.3); 2.5 mM MgCl_2_; 1 U AmpliTaq Gold polymerase (Applied Biosystems, Nieuwkerk a/d IJsel, The Netherlands) and 50 ng of template DNA. The PCR conditions were as follows: 94 °C for 10 min; 92 °C for 1 min, 60 °C for 1 min, 72 °C for 1 min (40 cycles); and 72 °C for 10 min.

All PCR products were purified with the MultiScreen HTS, 96 well Filtration System (Millipore, Carrigtwohill, Ireland). Subsequently, the purified products were sequenced using fluorescently labelled terminators (BigDye Terminators (v 1.1); Applied Biosystems, Foster City, CA, USA) with both M13-forward and M13-reverse sequencing primers. The sequencing products were analysed on an ABI 3730 DNA Analyser (Applied Biosystems) and the data analysis was performed using Sequencing Analysis Software Sequencing Analysis Software v5.3.1 with KBTM Basecaller. Sequence results were scored by visual inspection of the chromatograms (Applied Biosystems).

### Statistical analysis

We considered a discordance level of 5% or more to be clinically relevant, that is, leading to substantial change in routine clinical practice. To exclude such level of discordance under the assumption that the true discordance was 2.5% or less, we set the sample size at 304 paired samples. With this sample size, the precision in the estimated percentage of discordance was 2.5% (i.e., s.e. 1.25, half-width of the 95% confidence interval equal to 2.5%).

The comparison of patient and primary tumour characteristics between patients with *KRAS* wild-type and *KRAS* mutant primary tumours was done using Wilcoxon's rank sum test or *χ*^2^ for numerical or categorical variables, respectively. Differences in *KRAS* mutation status between the primary tumour and corresponding metastasis were analysed by calculating the percentage of concordance, and (clinically relevant) discordance, together with the corresponding Clopper–Pearson 95% confidence intervals. Differences were considered to be statistically significant when the *P*-value was below 0.05. All statistical tests were two-sided.

## Results

### Patient characteristics

We analysed *KRAS* codon 12 and 13 mutations in 320 matched primary colorectal tumours and liver metastases. The tumour cell percentages in all primary tumours and metastases were above 30%. We failed to obtain a *KRAS* mutation status in 15 patients; therefore our further analyses were performed in 305 paired samples. Patient characteristics are shown in Table 1.

### *KRAS* mutation and histopathological parameters

A total of 108 patients (35.4%) had a *KRAS* mutation in the primary tumour; of which 37 patients had a Gly12Asp mutation, 28 patients a Gly12Val mutation, 14 patients a Gly13Asp mutation, 10 patients a Gly12Cys mutation, 7 patients a Gly12Ser mutation, 7 patients a Gly12Ala mutation, 3 patients a Gly12Arg mutation, 1 patient a Gly12Asp and Gly12Ala mutation and 1 patient a Gly12Phe mutation (Table 2). Histopathological characteristics of the primary tumour were comparable between patients with and without a *KRAS* mutation (Table 1).

### Concordance of *KRAS* status in primary tumours and corresponding liver metastases

**Figure 1:**
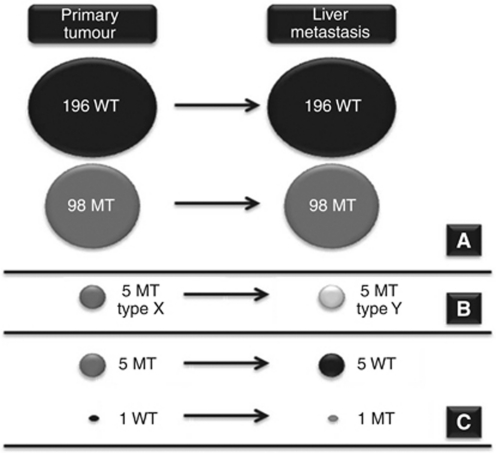
Overall concordance of the *KRAS* mutation status between primary tumour and liver metastasis (**A**), discordance without clinical impact (**B**), and discordance with clinical impact (**C**). Abbreviations: WT, wild type; MT, mutation.

In 294 patients (96.4% 95% CI 93.6–98.2%), the same *KRAS* mutation status was obtained from the primary tumour and the corresponding liver metastasis. In 11 patients (3.6% 95% CI 1.8–6.4%), of which 7 had synchronous metastases at diagnosis and 4 developed metachronous metastases, we found a discordance between primary tumours and metastases. Five patients had a *KRAS* mutation in the primary tumour and not in the liver metastasis. Only one patient had a wild-type status of the primary tumour, while the metastasis showed a *KRAS* mutation. In five patients, the primary tumours had different *KRAS* mutations compared with the metastases. One of these patients had two primary tumours. Both primary tumours had the same *KRAS* mutation (Gly13Asp), while the liver metastasis had a different *KRAS* mutation (Gly12Ser). In another patient, the primary tumour had a double mutation (Gly12Asp/Gly12Val) and the metastasis had a Gly12Asp mutation (Figure 1, Table 3). Taken together, the observed discordance was clinically relevant in only six patients (2.0% 95% CI 0.7–4.2%).

### Subsequent analyses in patients with a discordance of *KRAS* status

Several tests were performed to exclude bias of the test results. First, the HE coupes of all patients with a discordant *KRAS* mutation status between the primary tumour and liver metastasis were revised. The primary tumours and liver metastases had a mean tumour cell percentage of 65 and 60%, respectively. Subsequent independent reanalysis of the *KRAS* mutation status resulted in the same discordances.

Second, several mutation analyses were performed on different areas of the tumour and from different tumour blocks in order to establish possible tumour heterogeneity. Two patients showed heterogeneity of *KRAS* status within the primary tumour. One of these patients demonstrated two areas with a Gly12Asp mutation and one area with wild-type status, of which the latter resembled the liver metastasis. The other patient showed two different *KRAS* mutations within the same tumour, of which one is concordant with the liver metastasis (Table 3).

Third, 6 of the 11 patients with discordant results did have lymph nodes metastases at the time of diagnosis. *KRAS* mutation testing of all lymph nodes separately revealed overall concordant *KRAS* status between lymph node metastases and the primary tumour in three patients. The *KRAS* status of the lymph nodes in the other three patients showed heterogeneity, of which at least one lymph node metastases showed a different *KRAS* status compared with the primary tumour. However, this explains the discordance between the primary tumour and liver metastasis only in one patient (Table 3).

## Discussion

This is the first adequately powered study in CRC that compares *KRAS* mutation status between primary tumours and their corresponding liver metastases. We showed that tissue from the primary tumour can reliably be used for *KRAS* mutation testing in order to select patients for anti-EGFR therapy.

We observed a concordant *KRAS* mutation status in 96.4% of 305 paired samples of colorectal tumours and liver metastases. However, the difference in *KRAS* status was not clinically relevant in 5 of the 11 patients with discordant results, because both primary tumour and metastasis had a different *KRAS* mutation. Given the high statistical power of our analysis, we were able to obtain a highly accurate estimate of the level of discordance that enabled us to conclude that the level of discordance was 2.0%. The high rate of concordance is in agreement with the notion that *KRAS* mutations are considered as early driving events in CRC progression, and associated with the growth of small adenoma to clinically significant size (Vogelstein *et al*, 1988). Therefore, *KRAS* mutation status is expected to be equal in both primary tumours and metastases (Klein, 2009).

The previously reported lower concordance levels between primary tumours and metastases are most likely due to bias caused by false-negative results in underpowered studies. We calculated that 304 paired cases were needed to reliably exclude a rate of discordance of >5%, while previous studies included only 10 to 110 patients (Table 4). Moreover, in these studies metastases of different sites were compared with the primary tumour. As the molecular patterns may differ between metastatic sites (Klein, 2009), more reliable results are obtained when *KRAS* mutation status is tested more rigorously for each metastatic site. The liver is the predominant site of metastases in the majority of metastatic CRC patients; therefore the results of our large series of 305 liver metastases provide a solid reference for clinical decision making as to anti-EGFR therapy. Another issue is the fact that *KRAS* testing is technically not as straightforward as is often assumed. Several quality assurance systems are now in place, and the first ‘round robin’ test indicates that at least 30% of the experienced pathology laboratories fail to pass the threshold level of the quality assurance programs (Bellon *et al*, 2011). Other important facts about *KRAS* testing are the correct evaluation of the amount of tumour tissue in the sample and the sensitivity of testing methods. In a previous study, we demonstrated in >500 samples that both sequencing and real-time PCR are reliable methods (Tol *et al*, 2010).

A discordant *KRAS* status between the primary tumour and metastasis was observed in a small number of patients (3.6%). In these cases, tumour cells may have departed the primary lesions before the acquisition of a fully malignant phenotype to undergo somatic mutations or deletions at a distant site (Klein, 2009). Another explanation for the discordant results may be heterogeneity of *KRAS* status within the primary tumour, although this was the case in only a small number of patients. Finally, a discordance may in theory be explained by metastases from a non-detected second primary.

Previously published data showed that a considerable fraction (25%, Table 4) of colorectal lymph node metastases does not resemble the primary tumour in terms of *KRAS* mutation status. In 5 of the 25 lymph node metastases that we tested the *KRAS* status was not concordant with the primary tumour, which is consistent with the literature (Table 4). Therefore, lymph node metastases do not seem suitable for determination of the *KRAS* mutation status of colorectal carcinomas. Discordance in *KRAS* mutation status might be due to clonal selection during the process of metastasis, however, heterogeneity in lymph node metastases could explain this discordance in only one patient.

Eight different *KRAS* mutation types were observed in our study, of which Gly12Asp showed the highest frequency. Five patients (1.6%) harboured different *KRAS* mutation types in the primary tumour compared with the metastases. This confirms the findings of Cejas *et al* (2009) and Albanese *et al* (2004), who reported a small number of patients (4 and 7%, respectively) with different mutation types between primary tumours and metastases. A different *KRAS* mutation type between primary lung adenocarcinomas and corresponding lymph node metastases was also observed in only 1% of the patients (Schmid *et al*, 2009). Currently, all patients with a *KRAS* mutation are excluded from treatment with anti-EGFR antibodies, independently of the mutation type. However, a recent paper indicated that codon 13 mutated tumours may be sensitive to cetuximab treatment (De Roock *et al*, 2010). As we observed a low frequency in *KRAS* mutation type discrepancies between primaries and metastases, this is not of clinical importance in selecting patients for anti-EGFR therapy.

In conclusion, we demonstrated a high level of concordance of 96.4% between primary tumours and liver metastases, which for clinical purposes to select CRC patients for anti-EGFR therapy was even higher with 98%. The implication of these results for general oncology practice is that both tissue of primary tumour or liver metastasis may be used for *KRAS* mutation testing. The results of our study are only valid for liver metastases and cannot be extrapolated to other metastatic locations. Furthermore, we demonstrated that discordance of test results between primary tumour and metastases cannot account for the failure rate of anti-EGFR therapy in patients with *KRAS* wild-type tumours. Therefore, novel predictive markers in addition to *KRAS* and *BRAF* mutation status are warranted.

## Figures and Tables

**Table 1:** Distribution of tumour characteristics according to *KRAS* status of the primary tumour

	**Overall, *n*=305**	***KRAS* mutation, *n*=108**	***KRAS* wild type, *n*=197**	***P*-value**
*Age*				0.20
Median (IQR)	64 (57–70)	65 (58–71)	64 (57–70)	
*Gender*				0.37
Male	191 (62.6%)	64 (59.3%)	127 (64.5%)	
Female	114 (37.4%)	44 (40.7%)	70 (35.5%)	
*Metastases presentation*				0.45
Synchronous	169 (55.4%)	63 (58.3%)	106 (53.8%)	
Metachronous	136 (44.6%)	45 (41.7%)	91 (46.2%)	
*Tumour location*				0.63
Colon	167 (54.8%)	59 (54.6%)	108 (54.8%)	
Rectum	54 (17.7%)	16 (14.8%)	38 (19.3%)	
Rectosigmoid	80 (26.2%)	32 (29.6%)	48 (24.4%)	
Unknown	4 (1.3%)	1 (0.9%)	3 (1.5%)	
*Histopathological subtype*				0.12
Adenocarcinoma	271 (88.9%)	90 (83.3%)	181 (91.9%)	
Adenocarcinoma with muc. component	21 (6.9%)	10 (9.3%)	11 (5.6%)	
Mucinous adenocarcinoma	8 (2.6%)	5 (4.6%)	3 (1.5%)	
Unknown	5 (1.6%)	3 (2.8%)	2 (1.0%)	
*Differentiation grade*				0.21
Good	33 (10.8%)	13 (12.0%)	20 (10.2%)	
Moderate	196 (64.3%)	65 (60.2%)	131 (66.5%)	
Poor	52 (17.0%)	17 (15.7%)	35 (17.8%)	
Unknown	24 (7.9%)	13 (12.0%)	11 (5.6%)	
*T stage*				0.62
T1	4 (1.3%)	2 (1.9%)	2 (1.0%)	
T2	20 (6.6%)	9 (8.3%)	11 (5.6%)	
T3	231 (75.7%)	81 (75.0%)	150 (76.1%)	
T4	36 (11.8%)	11 (10.2%)	25 (12.7%)	
Unknown	14 (4.6%)	5 (4.6%)	9 (4.6%)	
*N stage*				0.10
N0	114 (37.4%)	46 (42.6%)	68 (34.5%)	
N1	87 (28.5%)	31 (28.7%)	56 (28.4%)	
N2	86 (28.2%)	26 (24.1%)	60 (30.5%)	
Unknown	18 (5.9%)	5 (4.6%)	13 (6.6%)	
*Number of lymph nodes examined*				0.28
Median (IQR)	10 (6–15)	10 (6–13)	10 (6–16)	
*Number of lymph node metastases*				0.15
Median (IQR)	1 (0–4)	1 (0–3)	1 (0–4)	

Abbreviation: IQR=interquartile range.

**Table 2:** Distribution of *KRAS* mutation types

**Codon 12/13**	**Patients with *KRAS* mutation (*n*, **%**)**
Gly12Asp	37 (34%)
Gly12Val	28 (26%)
Gly13Asp	14 (13%)
Gly12Cys	10 (9%)
Gly12Ser	7 (6%)
Gly12Ala	7 (6%)
Gly12Arg	3 (3%)
Gly12Phe	1 (1%)
Gly12Asp + Gly12Ala	1 (1%)

**Table 3:** Patients with a discordant *KRAS* status between primary tumour and liver metastasis. Multiple blocks of primary tumour tissue and lymph node metastases were tested when available

	***KRAS* status primary tumour**	***KRAS* status 2nd tumour**	***KRAS* status lymph node metastasis**	***KRAS* status liver metastasis**
1	Gly12Ala	—	LN 1: Gly12Ala	WT
			LN 2: Gly12Ala	
			LN 3: Gly12Ala	
2	Gly12Asp	—	—	WT
	Gly12Asp			
	WT			
3	Gly12Cys	—	—	WT
4	Gly12Asp	—	LN 1: Gly12Asp	WT
	Gly12Asp		LN 2: Gly12Asp	
	Gly12Asp		LN 3: Gly12Asp	
	Gly12Asp		LN 4: Gly12Asp	
			LN 5: WT	
5	Gly12Ser	—	—	WT
6	WT	—	—	Gly12Cys
7	Gly12Asp	—	LN 1: WT	Gly12Ala
			LN 2: WT	
			LN 3: WT	
8	Gly13Asp	Gly13Asp	LN 1: Gly13Asp	Gly12Ser
9	Gly12Ser	—	—	Gly12Ala
10	Gly12Cys	—	LN 1: Gly12Asp	Gly12Asp
	Gly12Asp		LN 2: Gly12Asp	
			LN 3: Gly12Asp	
			LN 4: Gly12Asp	
			LN 5: Gly12Asp	
			LN 6: WT	
11	Gly12Asp/Gly12Val	—	LN 1: Gly12Val	Gly12Asp
			LN 2: Gly12Val	
			LN 3: Gly12Val	
			LN 4: Gly12Asp	
			LN 5: Gly12Asp	
			LN 6: Gly12Asp	
			LN 7: Gly12Asp	

Abbreviation: WT=wild type.

**Table 4:** Overview of studies providing data on *KRAS* status of primary tumour and related metastasis

**Author study**	**Year**	**No. of pts**	**Analysed metastatic site**	**Method**	***KRAS* mutation in PT (%)**	***KRAS* mutation in PT, WT in M**	***KRAS* WT in PT, mutation in M**	**Total percentage of discordance**
Albanase	2004	30	Liver	SSCP analysis	14 (47%)	5/14 (36%)	4/16 (25%)	9/30 (30%)
Al-Mulla	1998	26	Liver	ASO/direct seq	10 (38%)	2/10 (20%)	3/16 (19%)	5/26 (19%)
		31	Lymph node	ASO/direct seq	10 (32%)	1/10 (10%)	5/21 (24%)	6/31 (19%)
Artale	2008	48	Diverse, 81% liver	Direct seq	11 (23%)	1/11 (9%)	2/37 (5%)	3/48 (6%)
Baldus	2010	20	Visceral metastasis	Direct seq	9 (45%)	1/9 (11%)	1/11 (9%)	2/20 (10%)
		55	Lymph node	Direct seq	29 (53%)	15/29 (52%)	2/26 (8%)	17/55 (31%)
Cejas	2010	93	Liver	Direct seq	30 (32%)	1/30 (3%)	4/63 (6%)	5/93 (5%)
		17	Lung	Direct seq	10 (59%)	1/10 (10%)	1/7 (14%)	2/17 (12%)
Etienne-Grimaldi	2008	48	Liver biopsy	PCR-RFLP	16 (33%)	0 (0%)	0 (0%)	0 (0%)
Italiano	2009	59	Not specified	Seq	23 (39%)	1/23 (4%)	2/36 (6%)	3/59 (5%)
Losi	1992	19	Local recurrence	Multiplex-ASPCR	12 (63%)	0 (0%)	0 (0%)	0 (0%)
		16	Metastasis, 38% liver	Multiplex-ASPCR	13 (81%)	0 (0%)	0 (0%)	0 (0%)
Loupakis	2009	43	Liver	Seq	Not mentioned	0 (0%)	2/^*^	2/43 (5%)
Molinari	2009	37	Diverse, 74% liver	Seq	16 (43%)	2/16 (13%)	1/21 (5%)	3/37 (8%)
		15	Lymph node	Seq	8 (53%)	0 (0%)	0 (0%)	0 (0%)
Oliveira	2006	28	Lymph node	Not mentioned	18 (64%)	2/18 (11%)	7/10 (70%)	9/28 (32%)
Oudejans	1991	31	Liver and lung	Hybridization	14 (45%)	1/14 (7%)	1/17 (6%)	2/31 (6%)
Perrone	2008	10	Diverse, mainly liver	Direct seq	2 (20%)	1/2 (50%)	1/8 (13%)	2/10 (20%)
Santini	2008	99	Diverse, 80% liver	Seq	38 (38%)	3/38 (8%)	1/61 (2%)	4/99 (4%)
Garm Spindler	2009	31	Not specified	qPCR	11 (35%)	2/11 (18%)	0/20 (0%)	2/31 (6%)
Suchy	1992	58	Autopsy material, not specified	Dot-blot hybridization	15 (26%)	0 (0%)	0 (0%)	0 (0%)
Weber	2006	36	Liver	Seq	14 (39%)	0 (0%)	0 (0%)	0 (0%)
Zauber	2003	42	Diverse, 93% lymph node, 5% liver	SCCP analysis + seq	22 (52%)	0 (0%)	0 (0%)	0 (0%)
								
Overall		892	All sites	All methods	345/849 (41%)	39/345 (11%)	35/504 (7%)	76/892 (9%)
		276	Liver	All methods	84/233 (36%)	8/84 (10%)	11/149 (7%)	21/276 (8%)
		129	Lymph nodes	All methods	65/129 (50%)	18/65 (28%)	14/64 (22%)	32/129 (25%)

Abbreviations: ASO=allele-specific oligonucleotide; ASPCR=allele-specific polymerase chain reaction; M=metastasis; pts=patients; PT=primary tumour; qPCR= quantitative PCR; RFLP=restriction fragment length polymorphism; SSCP=single strand conformational polymorphism; seq=sequencing. ^*^Total number of cases not specified.

## References

[bib1] Al-Mulla F, Going JJ, Sowden ET, Winter A, Pickford IR, Birnie GD (1998) Heterogeneity of mutant versus wild-type Ki-ras in primary and metastatic colorectal carcinomas, and association of codon-12 valine with early mortality. J Pathol 185: 130–138 9713338 10.1002/(SICI)1096-9896(199806)185:2<130::AID-PATH85>3.0.CO;2-M

[bib2] Albanese I, Scibetta AG, Migliavacca M, Russo A, Bazan V, Tomasino RM, Colomba P, Tagliavia M, La Farina M (2004) Heterogeneity within and between primary colorectal carcinomas and matched metastases as revealed by analysis of Ki-ras and p53 mutations. Biochem Biophys Res Commun 325: 784–791 15541358 10.1016/j.bbrc.2004.10.111

[bib3] Amado RG, Wolf M, Peeters M, Van Cutsem E, Siena S, Freeman DJ, Juan T, Sikorski R, Suggs S, Radinsky R, Patterson SD, Chang DD (2008) Wild-type KRAS is required for panitumumab efficacy in patients with metastatic colorectal cancer. J Clin Oncol 26: 1626–1634 18316791 10.1200/JCO.2007.14.7116

[bib4] Artale S, Sartore-Bianchi A, Veronese SM, Gambi V, Sarnataro CS, Gambacorta M, Lauricella C, Siena S (2008) Mutations of KRAS and BRAF in primary and matched metastatic sites of colorectal cancer. J Clin Oncol 26: 4217–4219 18757341 10.1200/JCO.2008.18.7286

[bib5] Baldus SE, Schaefer KL, Engers R, Hartleb D, Stoecklein NH, Gabbert HE (2010) Prevalence and heterogeneity of KRAS, BRAF, and PIK3CA mutations in primary colorectal adenocarcinomas and their corresponding metastases. Clin Cancer Res 16: 790–799 20103678 10.1158/1078-0432.CCR-09-2446

[bib6] Bellon E, Ligtenberg MJL, Tejpar S, Cox K, de Hertogh G, de Stricker K, Edsjö A, Gorgoulis V, Hoefler G, Jung A, Kotsinas A, Laurent-Puig P, López-Ríos F, Plato Hansen T, Rouleau E, Vandenberghe P, van Krieken JHJM, Dequeker E (2011) External quality assessment for *KRAS* testing is needed: set up of a European program and report of the first joined regional quality assessment rounds. Oncologist; doi:10.1634/theoncologist.2010-0429 10.1634/theoncologist.2010-0429 PMC3228116 21441573

[bib7] Benvenuti S, Sartore-Bianchi A, Di Nicolantonio F, Zanon C, Moroni M, Veronese S, Siena S, Bardelli A (2007) Oncogenic activation of the RAS/RAF signaling pathway impairs the response of metastatic colorectal cancers to anti-epidermal growth factor receptor antibody therapies. Cancer Res 67: 2643–2648 17363584 10.1158/0008-5472.CAN-06-4158

[bib8] Cejas P, Lopez-Gomez M, Aguayo C, Madero R, de Castro CJ, Belda-Iniesta C, Barriuso J, Moreno GV, Larrauri J, Lopez R, Casado E, Gonzalez-Baron M, Feliu J (2009) KRAS mutations in primary colorectal cancer tumors and related metastases: a potential role in prediction of lung metastasis. PLoS One 4: e8199 20020061 10.1371/journal.pone.0008199 PMC2792724

[bib9] De Roock W, Jonker DJ, Di Nicolantonio F, Sartore-Bianchi A, Tu D, Siena S, Lamba S, Arena S, Frattini M, Piessevaux H, Van Cutsem E, O’Callaghan CJ, Khambata-Ford S, Zalcberg JR, Simes J, Karapetis CS, Bardelli A, Tejpar S (2010) Association of KRAS p.G13D mutation with outcome in patients with chemotherapy-refractory metastatic colorectal cancer treated with cetuximab. JAMA 304: 1812–1820 20978259 10.1001/jama.2010.1535

[bib10] Di Nicolantonio F, Martini M, Molinari F, Sartore-Bianchi A, Arena S, Saletti P, De Dosso S, Mazzucchelli L, Frattini M, Siena S, Bardelli A (2008) Wild-type BRAF is required for response to panitumumab or cetuximab in metastatic colorectal cancer. J Clin Oncol 26: 5705–5712 19001320 10.1200/JCO.2008.18.0786

[bib11] Etienne-Grimaldi MC, Formento JL, Francoual M, Francois E, Formento P, Renee N, Laurent-Puig P, Chazal M, Benchimol D, Delpero JR, Letoublon C, Pezet D, Seitz JF, Milano G (2008) K-Ras mutations and treatment outcome in colorectal cancer patients receiving exclusive fluoropyrimidine therapy. Clin Cancer Res 14: 4830–4835 18676755 10.1158/1078-0432.CCR-07-4906

[bib12] Garm Spindler KL, Pallisgaard N, Rasmussen AA, Lindebjerg J, Andersen RF, Cruger D, Jakobsen A (2009) The importance of KRAS mutations and EGF61A>G polymorphism to the effect of cetuximab and irinotecan in metastatic colorectal cancer. Ann Oncol 20: 879–884 19179548 10.1093/annonc/mdn712

[bib13] Italiano A, Hostein I, Soubeyran I, Fabas T, Benchimol D, Evrard S, Gugenheim J, Becouarn Y, Brunet R, Fonck M, Francois E, Saint-Paul MC, Pedeutour F (2010) KRAS and BRAF mutational status in primary colorectal tumors and related metastatic sites: biological and clinical implications. Ann Surg Oncol 17: 1429–1434 20049644 10.1245/s10434-009-0864-z

[bib14] Karapetis CS, Khambata-Ford S, Jonker DJ, O’Callaghan CJ, Tu D, Tebbutt NC, Simes RJ, Chalchal H, Shapiro JD, Robitaille S, Price TJ, Shepherd L, Au HJ, Langer C, Moore MJ, Zalcberg JR (2008) K-ras mutations and benefit from cetuximab in advanced colorectal cancer. N Engl J Med 359: 1757–1765 18946061 10.1056/NEJMoa0804385

[bib15] Klein CA (2009) Parallel progression of primary tumours and metastases. Nat Rev Cancer 9: 302–312 19308069 10.1038/nrc2627

[bib16] Losi L, Benhattar J, Costa J (1992) Stability of K-ras mutations throughout the natural history of human colorectal cancer. Eur J Cancer 28A: 1115–1120 1627381 10.1016/0959-8049(92)90468-h

[bib17] Loupakis F, Pollina L, Stasi I, Ruzzo A, Scartozzi M, Santini D, Masi G, Graziano F, Cremolini C, Rulli E, Canestrari E, Funel N, Schiavon G, Petrini I, Magnani M, Tonini G, Campani D, Floriani I, Cascinu S, Falcone A (2009) PTEN expression and KRAS mutations on primary tumors and metastases in the prediction of benefit from cetuximab plus irinotecan for patients with metastatic colorectal cancer. J Clin Oncol 27: 2622–2629 19398573 10.1200/JCO.2008.20.2796

[bib18] Molinari F, Martin V, Saletti P, De Dosso S, Spitale A, Camponovo A, Bordoni A, Crippa S, Mazzucchelli L, Frattini M (2009) Differing deregulation of EGFR and downstream proteins in primary colorectal cancer and related metastatic sites may be clinically relevant. Br J Cancer 100: 1087–1094 19293803 10.1038/sj.bjc.6604848 PMC2669991

[bib19] Oliveira C, Velho S, Moutinho C, Ferreira A, Preto A, Domingo E, Capelinha AF, Duval A, Hamelin R, Machado JC, Schwartz Jr S, Carneiro F, Seruca R (2007) KRAS and BRAF oncogenic mutations in MSS colorectal carcinoma progression. Oncogene 26: 158–163 16953233 10.1038/sj.onc.1209758

[bib20] Oliveira C, Westra JL, Arango D, Ollikainen M, Domingo E, Ferreira A, Velho S, Niessen R, Lagerstedt K, Alhopuro P, Laiho P, Veiga I, Teixeira MR, Ligtenberg M, Kleibeuker JH, Sijmons RH, Plukker JT, Imai K, Lage P, Hamelin R, Albuquerque C, Schwartz Jr S, Lindblom A, Peltomaki P, Yamamoto H, Aaltonen LA, Seruca R, Hofstra RM (2004) Distinct patterns of KRAS mutations in colorectal carcinomas according to germline mismatch repair defects and hMLH1 methylation status. Hum Mol Genet 13: 2303–2311 15294875 10.1093/hmg/ddh238

[bib21] Oudejans JJ, Slebos RJ, Zoetmulder FA, Mooi WJ, Rodenhuis S (1991) Differential activation of ras genes by point mutation in human colon cancer with metastases to either lung or liver. Int J Cancer 49: 875–879 1959991 10.1002/ijc.2910490613

[bib22] Perrone F, Lampis A, Orsenigo M, Di Bartolomeo M, Gevorgyan A, Losa M, Frattini M, Riva C, Andreola S, Bajetta E, Bertario L, Leo E, Pierotti MA, Pilotti S (2009) PI3KCA/PTEN deregulation contributes to impaired responses to cetuximab in metastatic colorectal cancer patients. Ann Oncol 20: 84–90 18669866 10.1093/annonc/mdn541

[bib23] Santini D, Loupakis F, Vincenzi B, Floriani I, Stasi I, Canestrari E, Rulli E, Maltese PE, Andreoni F, Masi G, Graziano F, Baldi GG, Salvatore L, Russo A, Perrone G, Tommasino MR, Magnani M, Falcone A, Tonini G, Ruzzo A (2008) High concordance of KRAS status between primary colorectal tumors and related metastatic sites: implications for clinical practice. Oncologist 13: 1270–1275 19056857 10.1634/theoncologist.2008-0181

[bib24] Scaltriti M, Baselga J (2006) The epidermal growth factor receptor pathway: a model for targeted therapy. Clin Cancer Res 12: 5268–5272 17000658 10.1158/1078-0432.CCR-05-1554

[bib25] Schmid K, Oehl N, Wrba F, Pirker R, Pirker C, Filipits M (2009) EGFR/KRAS/BRAF mutations in primary lung adenocarcinomas and corresponding locoregional lymph node metastases. Clin Cancer Res 15: 4554–4560 19584155 10.1158/1078-0432.CCR-09-0089

[bib26] Suchy B, Zietz C, Rabes HM (1992) K-ras point mutations in human colorectal carcinomas: relation to aneuploidy and metastasis. Int J Cancer 52: 30–33 1500224 10.1002/ijc.2910520107

[bib27] Tol J, Dijkstra JR, Vink-Borger ME, Nagtegaal ID, Punt CJ, van Krieken JH, Ligtenberg MJ (2010) High sensitivity of both sequencing and real-time PCR analysis of KRAS mutations in colorectal cancer tissue. J Cell Mol Med 14: 2122–2131 19453520 10.1111/j.1582-4934.2009.00788.x PMC3823003

[bib28] Tol J, Koopman M, Cats A, Rodenburg CJ, Creemers GJ, Schrama JG, Erdkamp FL, Vos AH, van Groeningen CJ, Sinnige HA, Richel DJ, Voest EE, Dijkstra JR, Vink-Borger ME, Antonini NF, Mol L, van Krieken JH, Dalesio O, Punt CJ (2009) Chemotherapy, bevacizumab, and cetuximab in metastatic colorectal cancer. N Engl J Med 360: 563–572 19196673 10.1056/NEJMoa0808268

[bib29] Tol J, Punt CJ (2010) Monoclonal antibodies in the treatment of metastatic colorectal cancer: a review. Clin Ther 32: 437–453 20399983 10.1016/j.clinthera.2010.03.012

[bib30] Van Cutsem E, Kohne CH, Hitre E, Zaluski J, Chang Chien CR, Makhson A, D’Haens G, Pinter T, Lim R, Bodoky G, Roh JK, Folprecht G, Ruff P, Stroh C, Tejpar S, Schlichting M, Nippgen J, Rougier P (2009) Cetuximab and chemotherapy as initial treatment for metastatic colorectal cancer. N Engl J Med 360: 1408–1417 19339720 10.1056/NEJMoa0805019

[bib31] Vogelstein B, Fearon ER, Hamilton SR, Kern SE, Preisinger AC, Leppert M, Nakamura Y, White R, Smits AM, Bos JL (1988) Genetic alterations during colorectal-tumor development. N Engl J Med 319: 525–532 2841597 10.1056/NEJM198809013190901

[bib32] Weber JC, Meyer N, Pencreach E, Schneider A, Guerin E, Neuville A, Stemmer C, Brigand C, Bachellier P, Rohr S, Kedinger M, Meyer C, Guenot D, Oudet P, Jaeck D, Gaub MP (2007) Allelotyping analyses of synchronous primary and metastasis CIN colon cancers identified different subtypes. Int J Cancer 120: 524–532 17096353 10.1002/ijc.22343

[bib33] Zauber P, Sabbath-Solitare M, Marotta SP, Bishop DT (2003) Molecular changes in the Ki-ras and APC genes in primary colorectal carcinoma and synchronous metastases compared with the findings in accompanying adenomas. Mol Pathol 56: 137–140 12782759 10.1136/mp.56.3.137 PMC1187308

